# Carbon-encapsulated V_2_O_3_ nanorods for high-performance aqueous Zn-ion batteries

**DOI:** 10.3389/fchem.2022.956610

**Published:** 2022-09-02

**Authors:** Ziyi Hao, Weikang Jiang, Kaiyue Zhu

**Affiliations:** ^1^ State Key Laboratory of Catalysis, Dalian Institute of Chemical Physics, Chinese Academy of Sciences, Dalian, Liaoning, China; ^2^ Department of Chemistry, University of California, Los Angeles, Los Angeles, CA, United States; ^3^ Department of Chemical Physics, University of Science and Technology of China, Hefei, China

**Keywords:** carbon shell, V_2_O_3_ nanorods, phase transformation, V_2_O_5_·nH_2_O, aqueous Zn-ion batteries

## Abstract

Searching for stable cathodes is of paramount importance to the commercial development of low-cost and safe aqueous Zn-ion batteries (AZIBs). V_2_O_3_ is a good candidate for AZIB cathodes but has unsatisfied cycling stability. Herein, we solve the stability issue of a V_2_O_3_ cathode by coating a robust carbon shell. Strong evidence was provided that V_2_O_3_ was oxidized to favorable V_2_O_5_·nH_2_O during charging and the carbon shell could promote the oxidation of V_2_O_3_ to V_2_O_5_·nH_2_O. The discharge capacity was increased from ∼45 mA h g^−1^ to 336 mA h g^−1^ after V_2_O_3_ was oxidized to V_2_O_5_·nH_2_O, indicating a higher Zn^2+^-storage capability of V_2_O_5_·nH_2_O than V_2_O_3_. In addition, the rate-capability and long-term cycling performance are greatly enhanced after coating carbon shells on the surface of V_2_O_3_ nanorods. Therefore, the presented strategy of introducing carbon shells and fundamental insights into the favorable role of carbon shells in this study contribute to the advancement of highly stable AZIBs.

## 1 Introduction

In the past few decades, organic lithium-ion batteries (LIBs) have been widely used to power small-scale portable consumer electronics such as laptops and cellphones. However, further penetration of organic LIBs into the large-scale energy-storage (LSES) market is hindered by concerns over operational safety and cost, primarily due to the use of flammable organic electrolytes and expensive electrode materials (Tarascon and Armand, 2001; Yang et al., 2011). In contrast, aqueous LIBs attract much attention in terms of non-flammability, high ionic conductivity, and low cost, although they are characterized by low energy density and poor stability due to the narrow voltage window, high Li^+^ diffusion barriers, and the interference of unfavorable H^+^ intercalation (Bin et al., 2018; Wan et al., 2019; Posada-Pérez et al., 2021). As a promising alternative, rechargeable aqueous Zn-ion batteries (AZIBs) based on safe, non-toxic, and low-cost aqueous electrolytes are ideally suitable for LSES applications (Ming et al., 2019; Zhang N. et al., 2020; Chao et al., 2020). Moreover, compared to organic LIBs, the higher ionic conductivity (∼1 S cm^−1^ vs. ∼1–10 mS cm^−1^) and air tolerance of aqueous electrolytes enable a faster discharge/charge rate and easier assembly (Song et al., 2018; Blanc et al., 2020). Compared to aqueous LIBs, the high stability of zinc metal (Zn/Zn^2+^: 0.763 vs. standard hydrogen electrode) in water and air shows that Zn metal can be directly used as the anode of the AZIB, possessing high capacity and excellent cycling performance (Song et al., 2018; Wang F. et al., 2021; Wang X. et al., 2021).

Aqueous Zn-ion batteries (AZIBs), which consist of a Zn anode, a Zn^2+^/H^+^ storage cathode, and a Zn^2+^-salt electrolyte, hold great potential to meet the capacity, power, cost, and safety requirement of LSES owing to the following unique merits (Tang et al., 2019; Zhu et al., 2020b). First, the suitable redox potential (−0.76 V vs. standard hydrogen electrode (SHE)) of Zn/Zn^2+^ enables earth-abundant Zn metal to be directly used as the anode in AZIBs, thus contributing to a high theoretical capacity (820 mA h g^−1^/5,855 mA h cm^−3^). Second, the mildly near-neutral electrolyte (such as ZnSO_4_ and Zn(CF_3_SO_3_)_2_) can mitigate Zn-dendrite formation and endows ZIBs with good cycling stability. Moreover, good compatibility with water and air of Zn, together with the nontoxicity of the components enable ZIBs with facile fabrication and excellent recyclability.

Compared to the Zn plating/stripping at the anode, the unfavorable insertion/extraction behavior of Zn^2+^/H^+^ at the cathode determines the electrochemical properties (e.g., capacity and cycling stability) of AZIBs due to the strong electrostatic interactions between the divalent Zn^2+^ and the host lattice of cathode materials (Fang et al., 2018; Ming et al., 2018; Song et al., 2018; Liang et al., 2019; Jia et al., 2020). To address the issues, the recent development of AZIBs has been mostly focused on exploiting cathode materials with high capacity and cycling stability, together with elucidating the electrochemical mechanisms.

V-compounds are promising cathode materials for AZIBs because they feature unique open-layered or tunnel structures, which allow Zn^2+^/H^+^ or molecules to be inserted into the layers or tunnels (Ming et al., 2018; Wan and Niu, 2019; Zhu et al., 2019a; Zhu et al., 2019b; Zhang N. et al., 2020). Moreover, they have a wide range of electronic and crystallographic structures arising from various oxidation states (from V^5+^ to V^3+^) and coordination environments, thus, giving rise to high capacity. For example, α-V_2_O_5_ has been widely used as an AZIB cathode because a high theoretical capacity of 589 mA h g^−1^ is expected to be achieved *via* V^5+^/V^3+^ redox (Hu et al., 2017; Zhang et al., 2018; Zhou et al., 2018). Strong evidence was shown that V_2_O_5_·nH_2_O is the active material for the storage of Zn^2+^/H^+^ rather than anhydrous α-V_2_O_5_ in Zn||α-V_2_O_5_ batteries (Li et al., 2020; Zhu et al., 2021c). However, apart from the favorable phase transformation from α-V_2_O_5_ to V_2_O_5_·nH_2_O, anhydrous α-V_2_O_5_ undergoes severe dissolution in aqueous Zn^2+^-based solution both in the immersion state and during the charging/discharging process, resulting in poor long-term cycling stability (Zhu et al., 2021c). In addition, tunnel VO_2_ can deliver a high capacity of 322.6 mA h g^−1^ based on the V^3+^/V^4+^ redox process (Zhu et al., 2020a), whereas, after the *in situ* electrochemical oxidation of tunnel VO_2_ to layered V_2_O_5_·nH_2_O, the theoretical capacity is increased to 645.2 mA h g^−1^, benefiting from the additional contribution of V^4+^/V^5+^ redox process (Zhu et al., 2020a; Zhu et al., 2021b). Following the principle, if the V^5+^/V^3+^ redox process can be fully utilized in the V_2_O_3_ cathode, the theoretical capacity could reach 715 mA h g^−1^ (based on the mass of pristine V_2_O_3_).

In addition, V_2_O_3_ is far less harmful than other vanadium oxides as the V ion with a higher valence state is highly toxic. Therefore, V_2_O_3_ is a promising cathode material for AZIBs (Zhu et al., 2021a). V_2_O_3_ is a three-dimensional framework structure and consists of a mixture of a corner, edge, and face-sharing VO_6_ octahedral (Zhang S. et al., 2020). V_2_O_3_ delivers a very low capacity in AZIBs due to the inherently unsuitable structure and lack of active sites for Zn^2+^/H^+^-insertion (Luo et al., 2020; Li et al., 2021; Zhu et al., 2021a). However, Hu et al. reported that V_2_O_3_ delivers a high capacity of 350 mA h g^−1^ at 100 mA g^−1^ (Ding et al., 2019). The high capacity should result from the oxidation of V_2_O_3_ during charging. Then V_2_O_3_ was fully transformed into a high-active material (H-V_2_O_5_) through an *in situ* anodic oxidation strategy, finally achieving a capacity of 625 mA h g^−1^ at 0.1 A g^−1^ (Luo et al., 2020; Wang F. et al., 2021; Wang X. et al., 2021). It should be noted that V_2_O_3_ is also prone to dissolution in aqueous solutions and has low electric conductivity. The dissolution of V-based materials in aqueous solutions leads to a decrease in the capacity and poor cycling performance due to the loss of the active material, which should be addressed to achieve excellent performance (Zhang S. et al., 2020; Zhu et al., 2021b). The low electric conductivity limits the charge transfer rate and the specific capacity during cycling. To overcome the issues, *in situ* coating carbon shells on the V_2_O_3_ surface is a promising strategy. V_2_O_3_@C composites were synthesized by the pyrolysis of vanadium-based metal-organic frameworks (V-MOFs) or vanadium-based coordination polymers (V-CPs) (Li et al., 2014; Ding et al., 2019; Luo et al., 2020; Chen et al., 2021). Ren et al. constructed nitrogen-doped carbon-coated V_2_O_3_ (V_2_O_3_@N-C) by thermal treatment of V_2_O_5_@ZIF-8 composites in an inert atmosphere (Ren et al., 2022). It is to be noted that according to the aforementioned methods, the electric conductivity of V_2_O_3_ is greatly increased after introducing carbon, while the dissolution of V_2_O_3_ cannot be issued as the V_2_O_3_ particle cannot be fully and uniformly covered by a carbon layer.

Herein, we proposed a novel method for the *in situ* synthesis of V_2_O_3_@C nanorods. V_2_O_3_ nanorods were uniformly covered by a carbon shell using a PDA-assisted method inspired by the easy form of uniform PDA coating on various solid surfaces. Compared to V_2_O_3_, the electrical conductivity and stability in the AZIB for V_2_O_3_ @C are greatly improved due to the protection of the carbon shell. The resultant V_2_O_3_@C nanorod cathode delivers a high capacity of 290 mA h g^−1^ at 1 A g^−1^ and excellent rate capability (200 mA h g^−1^ at 20 A g^−1^). Furthermore, the Zn//V_2_O_3_@C cells show much better cycling stability at 1 and 10 A g^−1^ compared to Zn//V_2_O_3_. It is to be noted that V_2_O_3_ undergoes an oxidation transformation to V_2_O_5_·nH_2_O during the initial charging process. The carbon shell can promote the oxidation of V_2_O_3_ to V_2_O_5_·nH_2_O and regulate a favorable morphology of V_2_O_5_·nH_2_O, thus, enabling the high capacity and good stability of the Zn||V_2_O_3_@C batteries.

## 2 Experimental section

### 2.1 Synthesis of cathode materials

#### 2.1.1 Synthesis of VO_2_ nanorods

The VO_2_ nanorods were prepared through a hydrothermal method (Pan et al., 2012). In a typical procedure, 0.6 g V_2_O_5_ and 1.2 g H_2_C_2_O_4_·2H_2_O were first dissolved in 20 ml deionized (DI) water under vigorous stirring at 90°C for 2 h to form a blue solution. Then 4 ml of H_2_O_2_ (35 wt%) was added to the blue solution, followed by stirring the mixture for 30 min. Next, 50 ml ethanol was then added to the mixture and stirred for another 1 h. The formed dark-green mixture was subsequently loaded into a 100 ml autoclave with a Teflon liner and held at 170°C for 12 h. After that, the precipitate was collected and thoroughly washed with deionized water and ethanol and dried at 60°C for 12 h.

#### 2.1.2 Synthesis of VO_2_@polydopamine nanorods

A total of 1.0 g as-prepared VO_2_ nanorods were first well-dispersed in 200 ml tris(hydroxymethyl)aminomethane aqueous solution with a pH value of 8.5. Then, the solution was placed under an ultrasonic probe for ultrasonic dispersion for 30 min. Next, 125 mg dopamine hydrochloride was dissolved in the aforementioned dispersion solution and the mixture was stirred for 24 h for polymerization. After that, polydopamine-coated VO_2_ nanorods were washed using deionized water three times, separated by centrifugation, and then dried at 60°C for 12 h.

#### 2.1.3 Synthesis of V_2_O_3_@C nanorods

The as-prepared VO_2_@PDA powder was then calcined at 800°C for 3 h in a tube furnace under a 5% H_2_/Ar atmosphere, forming V_2_O_3_@C nanorods. For comparison, pure V_2_O_3_ was synthesized by directly calcining VO_2_ nanorods at 800°C for 3 h in a tube furnace under a 5% H_2_/Ar atmosphere.

### 2.2 Material characterization

#### 2.2.1 Phase and microstructure determination

X-ray diffraction (XRD) patterns of the samples were collected using a Rigaku D/MAX-2500/PC with Cu Kα radiation (*λ* = 1.54 Å at 40 kV and 200 mA). The data were recorded from 5° to 80° with an interval of 0.02° and a scan speed of 5° min^−1^. The morphologies of the samples were captured with the FEI Quanta 200 F. The crystalline structures and morphologies of the samples were also acquired with a high-resolution transmission electron microscope (HRTEM, JEM-ARM200F) operated at 300 kV. Elemental mapping along with the morphology was obtained by using a scanning transmission electron microscope (STEM, JEM-F200) equipped with an energy-dispersive X-ray spectrometer (EDS). Thermogravimetric analysis (TGA) was performed using a Pyris Diamond TG/DTA. Specimens were placed in an Al_2_O_3_ crucible with a lid, and TGA data were recorded under air with a flow rate of 50 ml min^−1^ while ramping from room temperature to 600°C at a rate of 2°C min^−1^, and then cooling naturally to room temperature.

#### 2.2.2 Surface chemistry

The surface chemical compositions and oxidation states of the elements were analyzed using a ThermoFisher Escalab 250 Xi + spectrometer with Al Ka X-ray radiation (hν = 1,486.6 eV). Before this analysis, the cycled electrodes were washed thoroughly with DI water to remove electrolyte residue and then dried in a glove box. All the binding energies were corrected by adventitious C 1s at 284.6 eV.

### 2.3 Electrochemical characterization

#### 2.3.1 Battery cell assembly

Electrochemical tests were carried out on CR2032-type coin cells. To prepare a cathode, 60 wt% active materials, 26 wt% Super-P, and 14 wt% polyvinylidene fluoride (PVDF) were thoroughly mixed and dispersed into N-Methyl pyrrolidone (NMP). The resultant slurry was then coated uniformly onto a 14 mm diameter stainless steel mesh, resulting in a ∼1.2 mg cm^−2^ active mass loading, followed by vacuum drying at 100°C for ∼12 h and compression at 10 MPa. In a full ZIB cell, zinc foil was used as the anode, 2 M ZnSO_4_ as the electrolyte, and glass microfiber filters (Whatman, Grade GF/A) as the separator.

#### 2.3.2 Electrochemical testing

The CR2032-type coin cells were assembled in the air and tested using a LAND battery testing system (CT 2001A) within a potential window of 0.2–1.6 V (vs. Zn/Zn^2+^). Cyclic voltammograms (CV) with V_2_O_3_@C as the working electrode and Zn metal as the counter and reference electrode were performed in aqueous ZnSO_4_ electrolytes within potential windows of 0.2–1.6 V using a Solartron 1,260/1,287 electrochemical workstation.

## 3 Results and discussion

### 3.1 Synthesis and characterizations of V_2_O_3_@C nanorods

**FIGURE 1:**
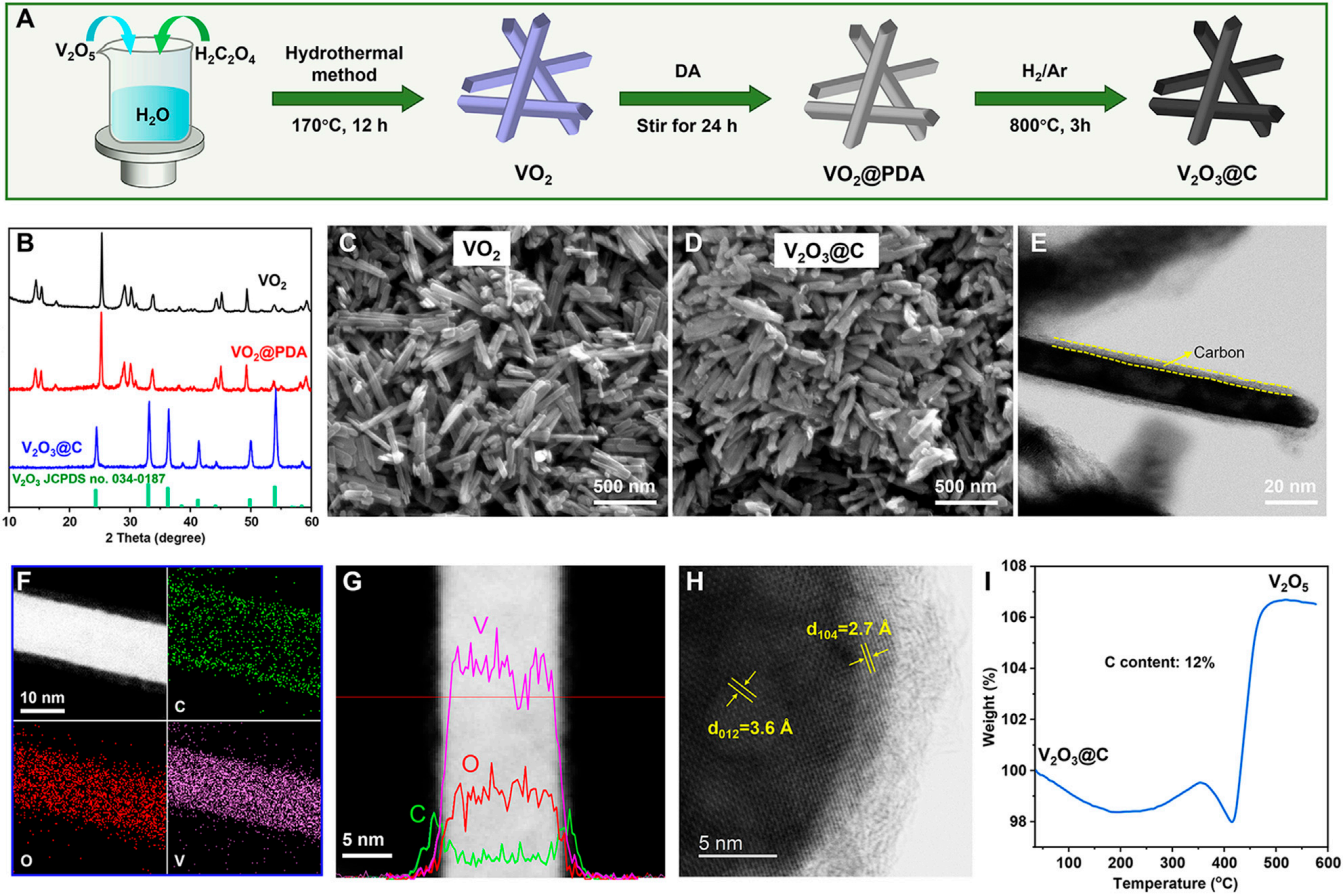
**(A)** Schematic illustration of the synthetic route for V_2_O_3_@C. **(B)** XRD patterns of as-prepared VO_2_, VO_2_@PDA, and V_2_O_3_@C. SEM images of **(C)** VO_2_ and **(D)** V_2_O_3_@C. **(E)** TEM, **(F)** STEM, and the corresponding elemental mapping of C, O, and V, **(G)** the linear elemental distribution, **(H)** HR-TEM image for V_2_O_3_@C nanorods. **(I)** TGA curves of V_2_O_3_@C nanorods under an air atmosphere.

The three-step preparation process of V_2_O_3_@C nanorods is schematically shown in Figure 1A. VO_2_ nanorods were first prepared by a hydrothermal method. Then, the polydopamine (PDA) was uniformly wrapped on the surface of VO_2_ nanorods due to the unique adhesion and reducibility (Lee et al., 2007; Liu et al., 2014). Finally, V_2_O_3_@C nanorods were obtained by calcinating VO_2_@PDA under 5% H_2_/Ar at 800°C for 3 h. Figures 1B and Supplementary Figure S1 show that the crystal structure of VO_2_ is well-maintained after introducing a PDA shell compared to pristine VO_2_. After reducing under 5% H_2_/Ar at 800°C, the PDA is reduced to carbon, while VO_2_ is reduced to V_2_O_3_, forming a core-shell structure of carbon-coated V_2_O_3_. XRD peaks of V_2_O_3_@C shown in Figure 1B are well indexed to standard V_2_O_3_ (JCPDS No. 71-0342). SEM and TEM images shown in Figures 1C–E reveal the nanorod morphology of V_2_O_3_@C with a width of ∼20 nm and length of ∼300 nm, similar to that of VO_2_. The scanning transmission electron microscopy (STEM) image and elemental mappings in Figure 1F show that V and O are uniformly distributed in the core while the shell is constituted by carbon. The linear elemental distribution in Figure 1G shows the higher concentration of V and O in the core along with a higher concentration of C in the shell, further demonstrating the core-shell structure of carbon-coated V_2_O_3_. The TEM images and elemental mapping shown in Figures 1E,F indicate that the thickness of the carbon shell is about 3 nm. The high-resolution TEM (HR-TEM) in Figure 1H shows that the lattice spacings of 3.6 Å and 2.7 Å in the core of the nanorods correspond well with the (012) and (104) of V_2_O_3_, respectively. Thermolgravimetric analysis (TGA) in Figure 1I suggests that the mass content of carbon in V_2_O_3_@C is 12%. It should be noted that the morphology and size of the nanorods are well-maintained during the introduction of the carbon layer. In contrast, pure VO_2_ nanorods were reduced to V_2_O_3_ particles with a diameter of ∼250 nm without introducing the PDA, as shown in Supplementary Figure S2. Therefore, the introduction of a carbon shell can prevent the growth of the nanorods during high-temperature treatment. To the best of our knowledge, this is the first study to use V_2_O_3_@C derived from PDA as the cathode of AZIBs.

### 3.2 Electrochemical performance of the V_2_O_3_@C cathode

**FIGURE 2:**
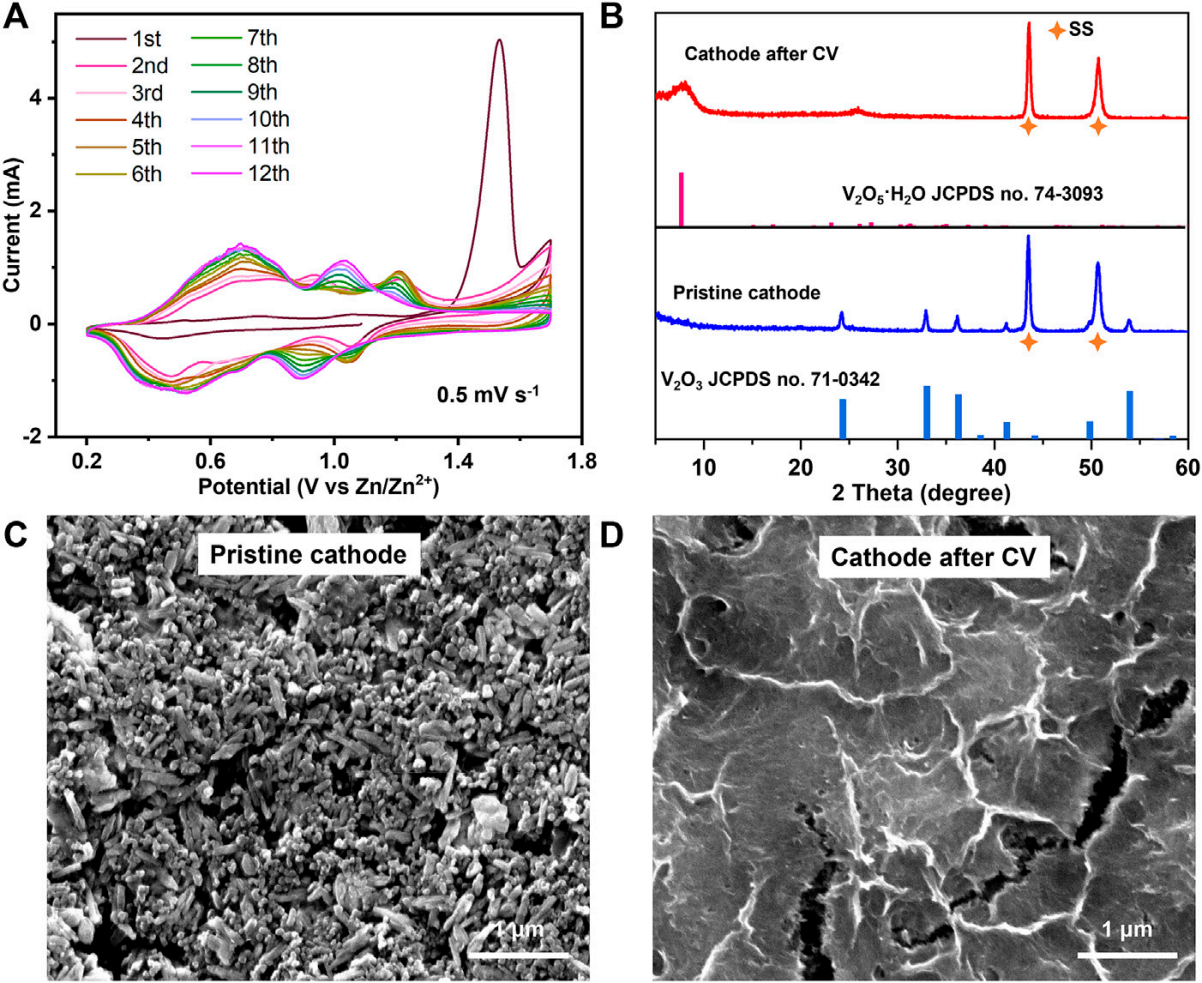
**(A)** CV curves of the V_2_O_3_@C cathode in 2 M ZnSO_4_. **(B)** XRD patterns and **(C,D)** SEM images of a pristine cathode and cathode after CV in Figure 2A for the V_2_O_3_@C cathode.

Figure 2A shows the CVs of the V_2_O_3_@C cathode in the first 12 cycles at a scan rate of 0.5 mV s^−1^. There is an obvious anodic peak at ∼1.53 V versus Zn/Zn^2+^ in the first cycle and the first-cycle CV is quite different from the following CVs, signaling that an irreversible phase transformation has occurred during the first CV cycle. The phase transformation is not fully completed after the first cycle as the following CV curves still slowly change with cycling and are finally stabilized after the 11th cycle. The CV curve of the 12th cycle is similar to that of hydrated V_2_O_5_·nH_2_O, inferring an irreversible phase transformation during cycling from V_2_O_3_ to V_2_O_5_·nH_2_O. A similar phase transformation has also been proved by previous references (Yan et al., 2018; Zhu et al., 2021b; Zhu et al., 2021d). V_2_O_3_ is a reductant and it will be oxidized at a high potential in an aqueous solution. In contrast, V_2_O_5_·nH_2_O is a strong oxidant. Therefore, the phase transformation from V_2_O_3_ to V_2_O_5_·nH_2_O is reasonable at a high potential in an aqueous solution. In addition, the CV curves of the V_2_O_3_@C cathode in 2 M ZnSO_4_ are similar to those of the V_2_O_3_@C cathode in 2 M Zn(OTf)_2_, indicating that the irreversible phase transformation of V_2_O_3_ to V_2_O_5_·nH_2_O also occurs in 2 M Zn(OTf)_2_. To unveil the transformation, XRD and SEM were performed. The XRD results in Figure 2B show that the V_2_O_3_ structure is fully transformed into V_2_O_5_·nH_2_O with low crystallinity, while the SEM images in Figures 2C,D indicate an obvious change in the morphology from nanorods to ultrathin nanowires. During the oxidation of V_2_O_3_, V_2_O_3_ undergoes the destruction of the crystal structure and recrystallization of V_2_O_5_·nH_2_O. Thus, the vanadium should be redistributed and located on the surface of the electrodes. Therefore, it is reasonable to conclude that the increase in the capacity and shape changes of CV curves are due to the transformation of V_2_O_3_ to V_2_O_5_·nH_2_O. It is worth noting that even if a carbon shell is coated on the V_2_O_3_ surface, the V species is also transferred from the inside of the carbon shell to the outside during oxidation. The results indicate that the carbon shell is porous, and thus, permeable to the electrolyte solution. The porous structure of the carbon shell can be further confirmed by a much higher BET area and smaller pore size of V_2_O_3_@C (36 m^2^ g^−1^, 14 nm) compared to V_2_O_3_ (10 m^2^ g^−1^, 28 nm), as shown in Supplementary Figure S4.

**FIGURE 3:**
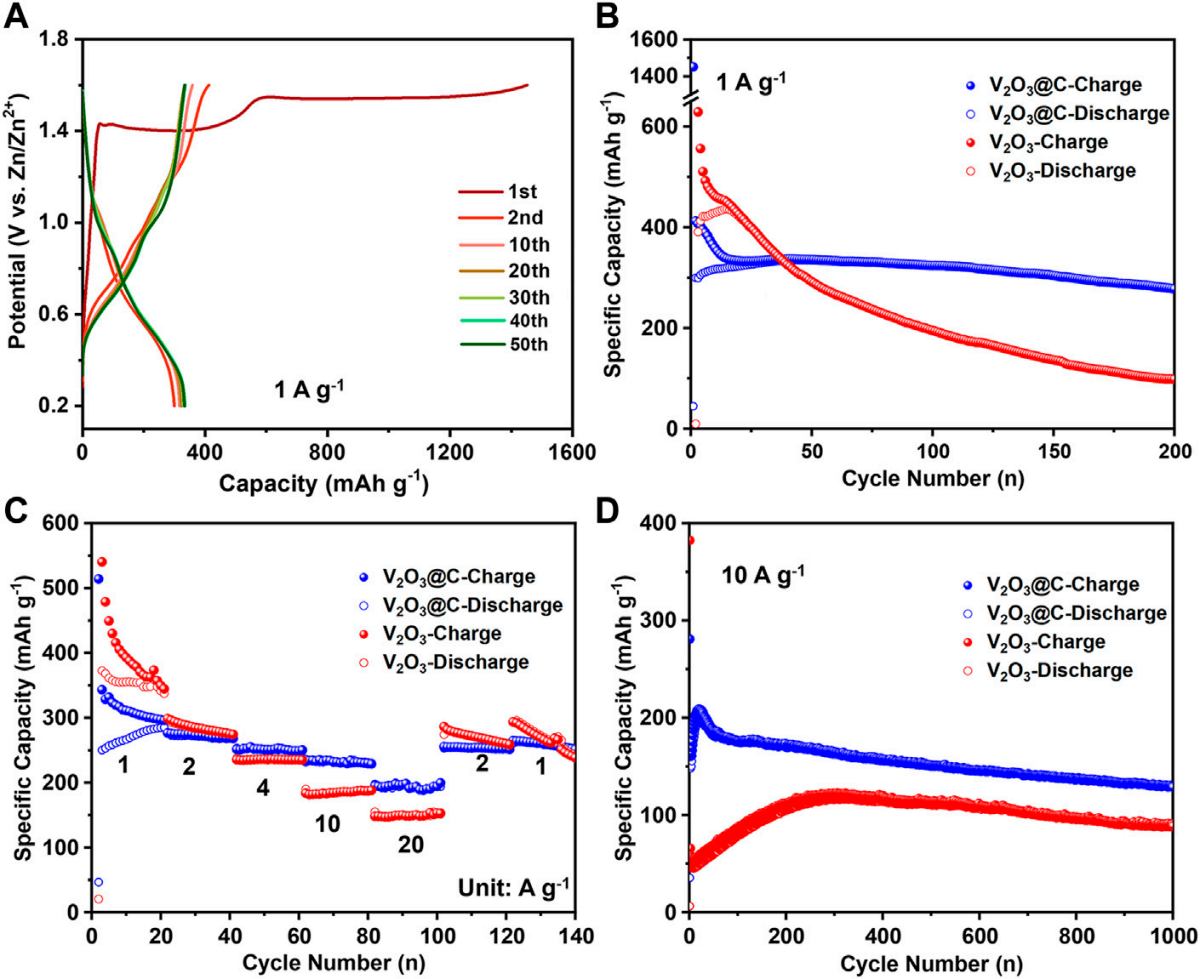
**(A)** Discharge/charge curves represent cycles at 1 A g^−1^ for the V_2_O_3_@C cathode in aqueous ZnSO_4_ solution. **(B)** cycling performance at a low rate (1 A g^−1^), **(C)** rate performance, and **(D)** cycling performance at a high rate (10 A g^−1^) for V_2_O_3_ and V_2_O_3_@C cathodes in aqueous ZnSO_4_ solution.

Figure 3 shows the electrochemical performance of the V_2_O_3_@C cathode in AZIBs. The discharge/charge curves in Figure 3A show that the first discharge capacity is only 45 mA h g^−1^ at 1 A g^−1^, while the first charge capacity is as high as 1,450 mA h g^−1^ with two plateaus at ∼1.4 and ∼1.5 V, respectively. The latter is a sign of the phase transformation from V_2_O_3_ to V_2_O_5_·nH_2_O when charging. The higher charge capacity than the theoretical capacity (715 mA h g^−1^) arises from the low Faraday efficiency of the oxidation reaction. After the oxidation at the first cycle, the discharge capacity of the second cycle is increased to 300 mA h g^−1^, while the charge capacity is decreased to 412 mA h g^−1^. Then the discharge capacity is slowly increased and stabilized at 336 mA h g^−1^, while the charge capacity is decreased slowly to 335 mA h g^−1^. The low Coulombic efficiencies in the initial cycles are because there are some side reactions during the electro-oxidization of V_2_O_3_ to V_2_O_5_·1.75H_2_O. It should be noted that the aforementioned capacity is based on the initial mass of V_2_O_3_. Suppose all V_2_O_3_ is fully transformed to V_2_O_5_·1.75H_2_O [the water content is obtained in the d-spacing of (001)], 1 g V_2_O_3_ would be transformed to 1.37 g V_2_O_5_·1.75H_2_O. Therefore, the capacity of 336 mA h g^−1^ based on the V_2_O_3_ mass corresponds to 245 mA h g^−1^ based on the V_2_O_5_·1.75H_2_O mass. The results are consistent with the CV results (Figure 2A) that V_2_O_3_ undergoes fast and incomplete oxidation in the first cycle, followed by slow and complete oxidation. Figure 3B shows that the capacity retention rate is 83% after 200 cycles at 1 A g^−1^ after full oxidation of V_2_O_3_ to V_2_O_5_·nH_2_O in the V_2_O_3_@C cathode, demonstrating excellent long-term cycle stability. It should be noted that the V_2_O_3_@C cathode shows much better stability than the V_2_O_3_ cathode, as shown in Figure 3B. Additionally, Figures 3C,D indicate that the rate capability and long-term stability at 10 A g^−1^ of the V_2_O_3_@C cathode are also superior to that of the V_2_O_3_ cathode. Thus, the carbon shell should play a vital role in long-term stability. It should be noted that the specific capacity of the V_2_O_3_@C cathode is based on the total mass of V_2_O_3_ and C, leading to a lower capacity of the V_2_O_3_@C cathode than that of the V_2_O_3_ cathode. Although the capacities of the first 25 cycles for the V_2_O_3_@C cathode are a little lower than those for the V_2_O_3_ cathode at 1 A g^−1^, the former shows a much higher capacity than the latter after 25 cycles (see Figure 3B). Furthermore, Figure 3D shows that the V_2_O_3_@C cathode delivers a much higher capacity (200 mA h g^−1^) than the V_2_O_3_ cathode (150 mA h g^−1^) at a high current density of 20 A g^−1^. Figure 3C also shows 94 and 93% capacity recovery after the current density resumes to 2 and 1 A g^−1^ from 20 A g^−1^ excursion. At 10 A g^−1^, 65% of the highest capacity (205 mA h g^−1^) is retained after 1,000 cycles, as shown in Figure 3D. These results suggest that the V_2_O_3_@C cathode possesses good cycling stability benefiting from the favorable role of the carbon shell. At 1 A g^−1^ and 10 A g^−1^, it takes fewer cycles (or time) to achieve the highest discharge capacity for the V_2_O_3_@C cathode compared to the V_2_O_3_ cathode (see Figures 3B–D), indicating that the carbon shell could promote the oxidation of V_2_O_3_. Intrinsically, V_2_O_3_ has poor Zn^2+^-storage ability, far worse than V_2_O_5_·nH_2_O. Thus, AZIBs will deliver the highest discharge capacity when V_2_O_3_ is fully transformed to hydrated V_2_O_5_·nH_2_O. Therefore, it is reasonable to say that carbon promotes the oxidation of V_2_O_3_ to V_2_O_5_·nH_2_O benefiting from the high conductivity and small particle size of V_2_O_3_ in V_2_O_3_@C.

Overall, the carbon shell in V_2_O_3_@C has the following roles. First, the carbon shell prevents the growth of V_2_O_3_ during preparation at high temperatures (800°C) as shown in Figures 1D and Supplementary Figure S2. Second, the carbon shell should decrease the dissolution of V_2_O_3_ and increases the electronic conductivity of V_2_O_3_ arising from the outer layer protection. Third, the carbon shell carbon promotes the oxidation of V_2_O_3_ to V_2_O_5_·nH_2_O.

### 3.3 The *ex situ* characterization and reaction mechanism

**FIGURE 4:**
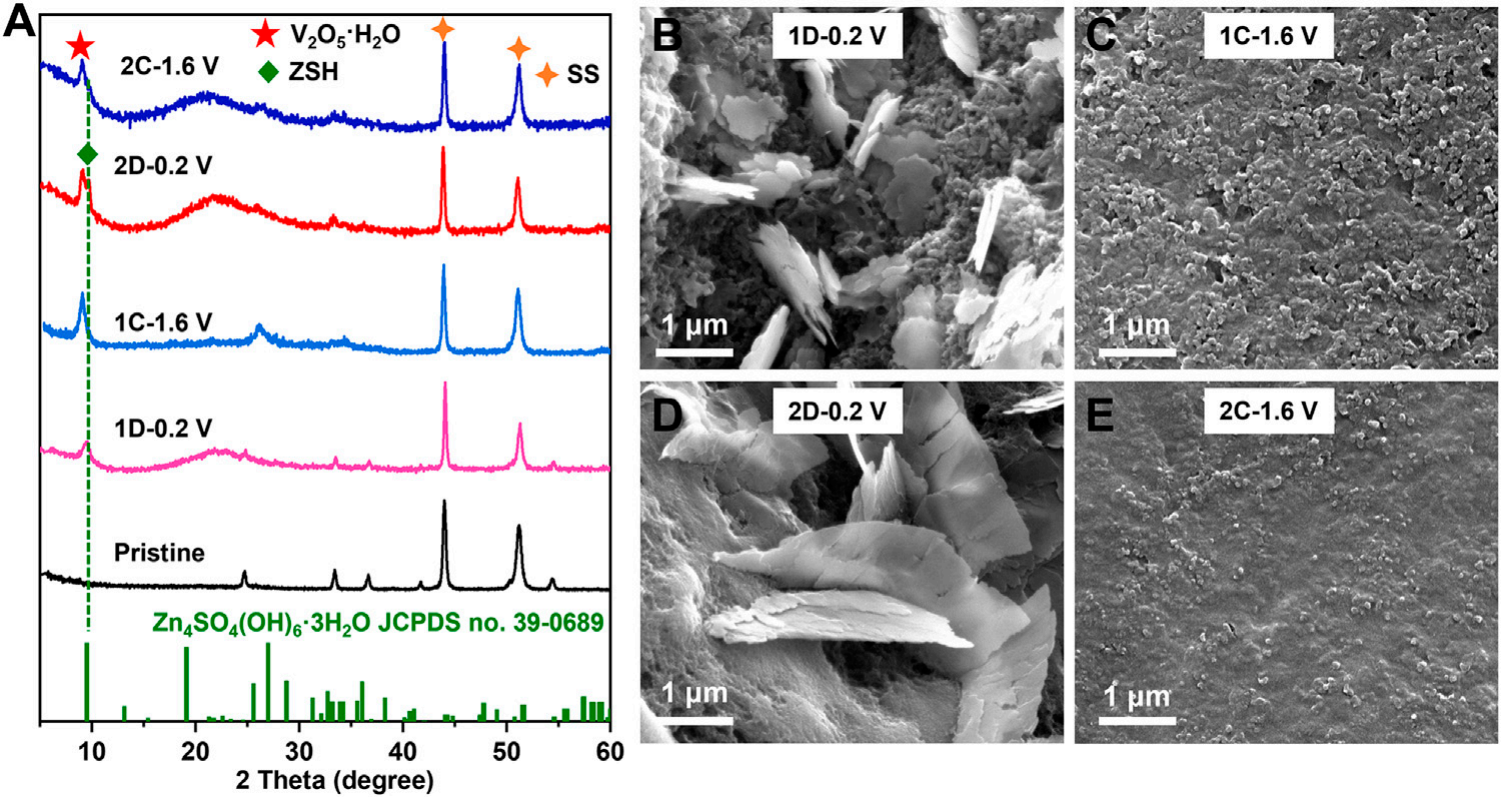
**(A)** XRD patterns of the V_2_O_3_@C cathode at pristine state (Pristine), discharged state in the first or second cycle (1D-0.2 V; 2D-0.2 V), charged state in the first cycle or second cycle (1C-1.6V; 2C-1.6 V) in aqueous ZnSO_4_ solution at 0.5 A g^−1^. SEM images of the V_2_O_3_@C cathode at **(B)** discharged state at the first cycle (1D-0.2 V), **(C)** charged state in the first cycle (1C-1.6 V), **(D)** discharged state in the second cycle (2D-0.2 V), and **(E)** charged state in the second cycle (2C-1.6 V), in aqueous ZnSO_4_ solution at 0.5 A g^−1^.

To further confirm the transformation of V_2_O_3_ to V_2_O_5_·nH_2_O and the Zn^2+^-storage mechanism of the *in situ* generated V_2_O_5_·nH_2_O cathode, *ex situ* XRD and SEM were performed during cycling. Figure 4A shows that a weak peak corresponding to Zn_4_SO_4_(OH)_6_·3H_2_O (ZSH) appears when discharged to 0.2 V (1D-0.2 V); upon charging to 1.6 V (1C-1.6 V), the peak corresponding to ZSH completely disappeared. According to previous studies, the root cause of ZSH formation is H^+^ intercalation (Huang et al., 2018; Zhu et al., 2021d). Additionally, the XRD peaks (Figure 4A) of V_2_O_3_ after the first cycle in the aqueous ZnSO_4_ (2 M) electrolyte vanished, probably due to the phase transformation. After the complete oxidation of V_2_O_3_ to V_2_O_5_·nH_2_O, the XRD peak for ZSH also appears reversible and vanishes during cycling, indicating the reversible H^+^ intercalation/de-intercalation into/from V_2_O_5_·nH_2_O. The SEM images in Figures 4B–E show that ZSH nanoflakes appear on the surface of V_2_O_3_ and V_2_O_5_·nH_2_O electrodes upon discharge and disappear after charge.

**FIGURE 5:**
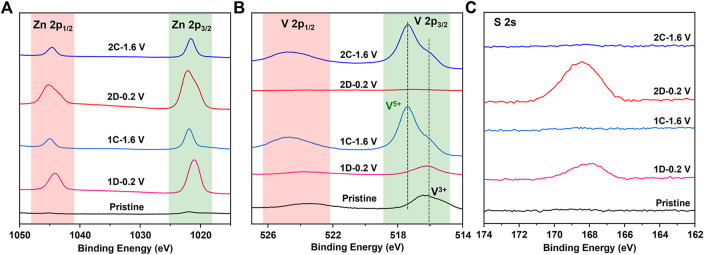
XPS spectra of **(A)** Zn 2p, **(B)** V 2p, and **(C)** S 2s for the V_2_O_3_@C cathode at pristine state (Pristine), discharged state in the first or second cycle (1D-0.2 V; 2D-0.2 V), and charged state in the first cycle (1C-1.6V; 2C-1.6 V) in aqueous ZnSO_4_ solution at 0.5 A g^−1^.

The aforementioned results are also confirmed by XPS in Figure 5, where a sharper increase in Zn 2p intensity is observed on the fully discharged cathode for V_2_O_3_ (1D-0.2 V) and V_2_O_5_·nH_2_O (2D-0.2 V) than those on the pristine and charged states (1C-1.6 V and 2C-1.6 V), suggesting that a possible Zn-containing compound (ZSH) is formed on the surface of the electrode. It is to be noted that XPS is a surface technique capable of penetrating ∼5 nm depth. Therefore, the much lower V signals on the XPS spectra of the discharged products (and 2D-0.2 V) compared to the pristine and the charged electrodes (1C-1.6 V and 2C-1.6 V) could be due to the interference of surface ZSH. The extremely low V signals of the discharged V_2_O_5_·nH_2_O electrode (2D-0.2 V) infer that there is a lot of surface ZSH covered on the electrode. Therefore, *ex situ* SEM, XRD, and XPS have convincingly demonstrated that H^+^ indeed takes part in the electrochemical process during cycling.

**FIGURE 6:**
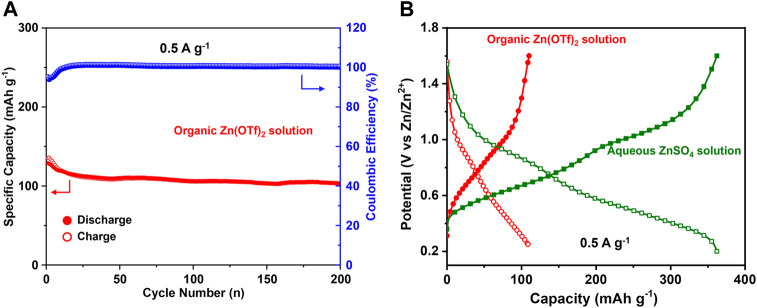
**(A)** Cycling performance of the V_2_O_3_@C cathode in organic Zn(OTf)_2_ solution at 0.5 A g^−1^. **(B)** Discharge/charge curves in the 50th cycle for the V_2_O_3_@C cathode in organic Zn(OTf)_2_ solution and aqueous ZnSO_4_ solution at 0.5 A g^−1^.

Additionally, the electrochemical performance of the oxidized electrode (2C-1.6 V) in the aqueous ZnSO_4_ (2 M) solution and organic Zn(CF_3_SO_3_)_2_ (1 M) solution (solvent: acetonitrile) was compared to demonstrate the Zn^2+^-intercalation. Figure 6 shows that the capacity in organic Zn(OTf)_2_ solution is only ∼110 mA h g^−1^ at a current of 0.5 A g^−1^, much lower than that (∼360 mA h g^−1^) in aqueous ZnSO_4_ solution. As there is no H^+^ in the organic Zn(OTf)_2_ solution, only Zn^2+^ can be involved during cycling. The capacity in organic Zn(OTf)_2_ solution should be attributed to Zn^2+^-intercalation/deintercalation. The higher capacity in aqueous ZnSO_4_ solution than that in organic Zn(OTf)_2_ solution also infers the involvement of H^+^ in aqueous ZnSO_4_ solution. Overall, apart from the involvement of Zn^2+^, H^+^ also takes part in the electrochemical process during cycling.

**FIGURE 7:**
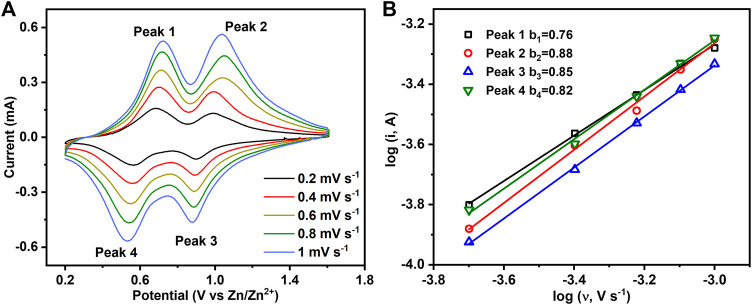
**(A)** CV curves of hydrated V_2_O_5_·nH_2_O obtained by the electrochemical oxidation of V_2_O_3_ at different scan rates. **(B)** Plots of log(i) vs. log(v) for the four redox peaks in Figure 7A.

To further understand the intercalation behavior of Zn^2+^/H^+^, CV measurements at different scan rates from 0.2 to 1 mV s^−1^ were performed on hydrated V_2_O_5_·nH_2_O obtained by the electrochemical oxidation of V_2_O_3_ (Figure 7A). There are two pairs of redox peaks corresponding to the changes in the oxidation state of V^5+^/V^4+^ (peaks 2 and 3) and V^4+^/V^3+^ (peaks 1 and 4). Generally, the peak current (i) of CVs can be related to the scan rate (ʋ) by the following empirical power-law relationship (Augustyn et al., 2014):

$$i=k_{1}v+k_{2}v^{1/2}\approx av^{b},$$

where k_1_, k_2_, a, and b are variable parameters with b = 0.5 for a diffusion-controlled charge-transfer process and 1.0 for a surface-controlled capacitive process. Figure 7B shows that b-values obtained from the slopes of log(i) versus log(ʋ) for the four peaks are 0.76, 0.88, 0.85, and 0.82. Therefore, the intercalation behavior of Zn^2+^/H^+^ in hydrated V_2_O_5_·nH_2_O obtained by the electrochemical oxidation of V_2_O_3_ is controlled by ionic diffusion and surface capacitance synchronously.

## 4 Conclusion

In this study, carbon shell-coated V_2_O_3_ nanorods were successfully prepared and showed good electrochemical performance in ZIBs. Strong pieces of evidence were provided to demonstrate that V_2_O_3_ would be oxidized to V_2_O_5_·nH_2_O when charged, while the carbon shell could promote the oxidation of the V_2_O_3_ core and have a favorable role in stability. Benefiting from the carbon shell, V_2_O_3_@C exhibits a much improved rate-capability and cycling stability compared to pure V_2_O_3_. The discharge capacity of V_2_O_3_ was only ∼45 mA h g^−1^ at 1 A g^−1^, while the capacity was increased to 350 mA h g^−1^ after oxidizing V_2_O_3_ to V_2_O_5_·nH_2_O. When the current is increased from 1 to 20 A g^−1^, the capacity retention for the V_2_O_3_@C electrode is 69%, much higher than that for V_2_O_3_ electrode (44%). Additionally, the long-term stabilities at the currents of 1 A g^−1^ and 10 A g^−1^ for V_2_O_3_@C are much better than those for the V_2_O_3_ electrode. Overall, coating the carbon shell on the active materials provides a way to enhance the rate-capability and long-term cycling stability, and the strategy of introducing the carbon shell on the surface of V_2_O_3_ nanorods can be extended to other materials.

## Data Availability

The original contributions presented in the study are included in the article/Supplementary Material; further inquiries can be directed to the corresponding author.

## Supplementary material

Supplementary Figure S1 XRD patterns of as-prepared VO_2_,VO_2_@PDA, and standard VO_2_.

Supplementary Figure S2 XRD patterns of VO_2_ and V_2_O_3_ prepared without using PDA.

Supplementary Figure S3 CV curves of the V_2_O_3_@C cathode in 2 M Zn(OTf)_2_.

Supplementary Figure S4 Nitrogen adsorption–desorption isotherm curves of V_2_O_3_ and V_2_O_3_@C powders. Inset shows the BET area and average pore size.
