# Supplementary material for: Carbon-encapsulated V2O3 nanorods for high-performance aqueous Zn-ion batteries
Source: Front Chem. 2022 Sep 2;10:956610. doi: 10.3389/fchem.2022.956610 (PMC9479548; doi:10.3389/fchem.2022.956610)
Supplement: Supplementary file 1 [file Presentation1.pdf]

Supplementary Material

Carbon Encapsulated V_2O_3 Nanorods for High-Performance Aqueous Zn-Ion Batteries

Ziyi Hao[1,2], Weikang Jiang[1,3], Kaiyue Zhu[1*]

[1]State Key Laboratory of Catalysis, Dalian Institute of Chemical Physics, Chinese Academy of Sciences, Dalian 116023, Liaoning, China

[2]Department of Chemistry, University of California, Los Angeles, CA 90095, United States

[3]Department of Chemical Physics, University of Science and Technology of China, Anhui 230026, Hefei, China

*** Correspondence:**
Corresponding Author
zky218@dicp.ac.cn

1 Supplementary Figures

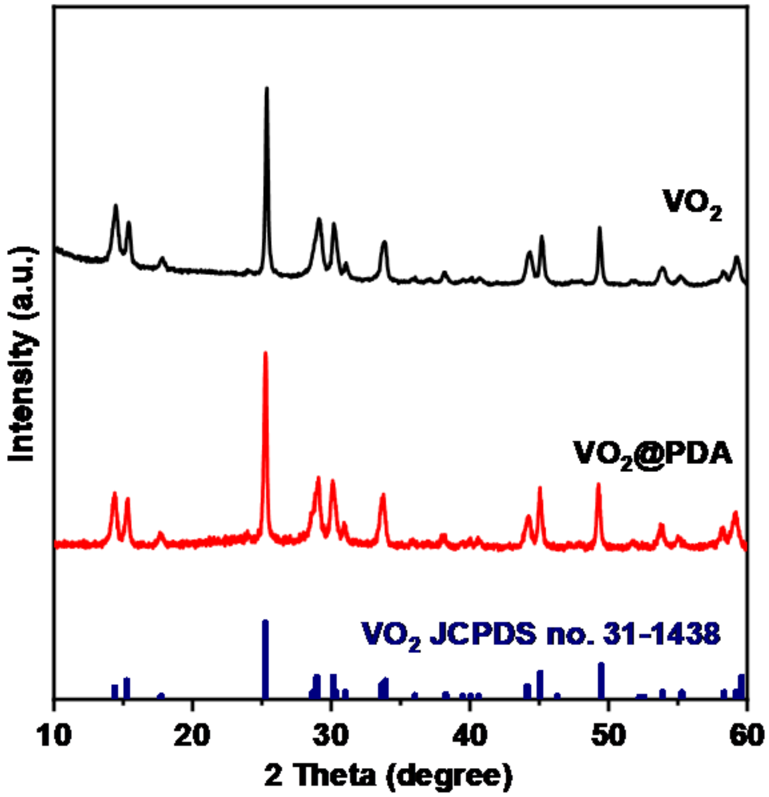

Supplementary Figure 1. XRD patterns of as-prepared VO_2,VO_2@PDA, and standard VO_2.

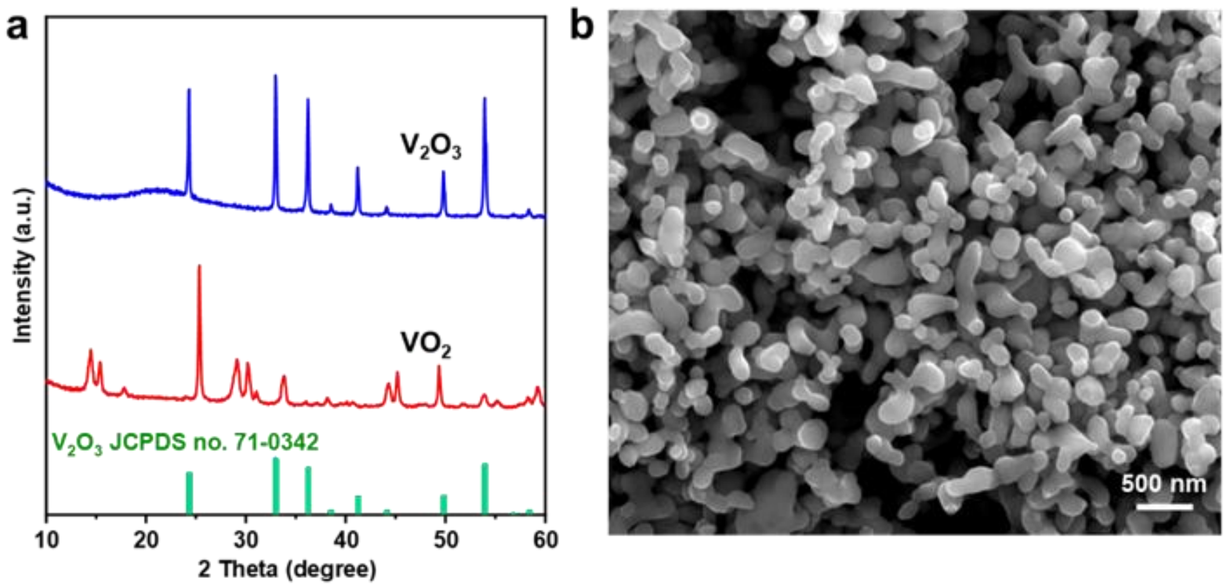

Supplementary Figure 2. XRD patterns of VO_2 and V_2O_3 prepared without using PDA.

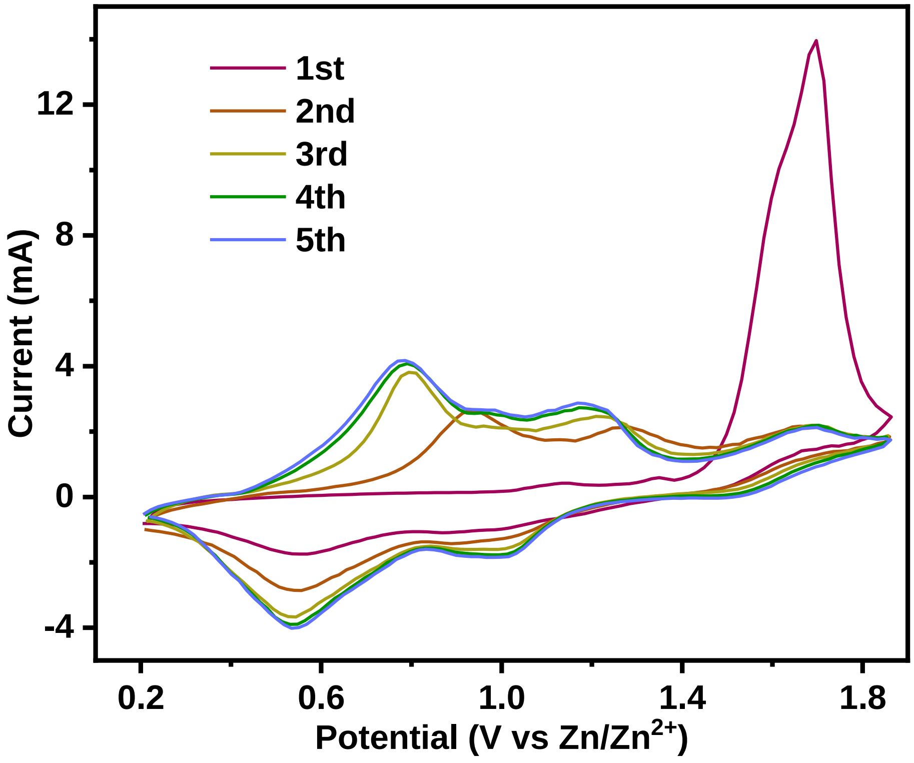

Supplementary Figure 3. CV curves of V_2O_3@C cathode in 2 M $Zn(OTf)_2$.

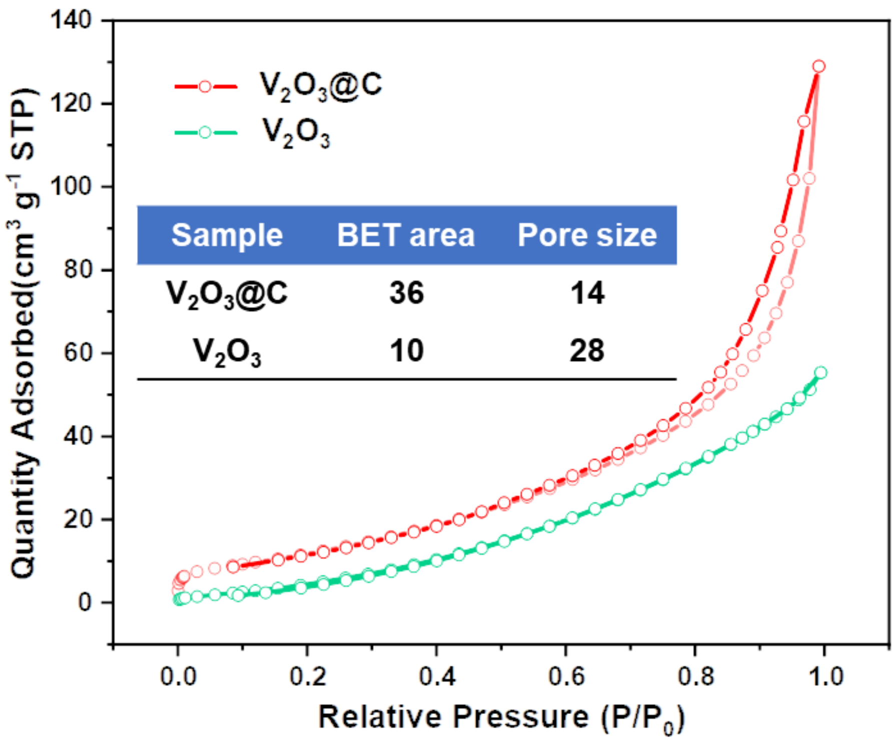

Supplementary Figure 4. Nitrogen adsorption-desorption isotherm curves of V_2O_3 and V_2O_3@C powders. Inset shows the BET area and average pore size.
